# Surveillance of In Vitro Activity of Cefiderocol Against Carbapenem-Resistant Gram-Negative Clinical Isolates in a Tertiary Care Hospital

**DOI:** 10.7759/cureus.67164

**Published:** 2024-08-19

**Authors:** Chakrapani Kammineni, Sreeja Vamsi, Sreekanth Reddy Basireddy

**Affiliations:** 1 Microbiology, Government Medical College, Adoni, IND; 2 Microbiology, SVS Medical College and Hospital, Mahabubnagar, IND; 3 Microbiology, Government Medical College, Kadapa, IND; 4 Microbiology, Siriraj Hospital, Mahidol University, Bangkok, THA

**Keywords:** colistin, carbapenem resistant gram-negative bacteria, resistance surveillance, mic testing, disc diffusion, cefiderocol

## Abstract

Introduction: Antibiotic resistance among Gram-negative bacterial isolates is increasingly observed. With the emergence of carbapenem-resistant and pan-resistant pathogens, treating these resistant infections is becoming more challenging due to the limited number of effective drugs. There is a desperate need for the discovery of new antibiotics with novel mechanisms of action. Cefiderocol is one such novel antibiotic with a unique siderophore-based mechanism of action, which has been recently approved for clinical use against drug-resistant pathogens. The present study aims to identify the in vitro activity of cefiderocol against major carbapenem-resistant clinical isolates, including those resistant to colistin.

Materials and methods: One hundred and one carbapenem-resistant clinical isolates were included in the study. Identification and antibiotic susceptibility testing were performed using the automated VITEK® 2 Compact (bioMérieux SA, Marcy-l'Étoile, France) identification and susceptibility testing system, except for colistin and cefiderocol. Colistin resistance in Enterobacterales and *Pseudomonas aeruginosa* was assessed using the agar dilution minimum inhibitory concentration method, while for* Acinetobacter baumannii*, broth microdilution method was employed. Cefiderocol susceptibility testing was conducted using the Kirby-Bauer disc diffusion method with 30 µg discs on standard Mueller-Hinton agar plates. For selected isolates, cefiderocol minimum inhibitory concentration detection was performed using broth microdilution with iron-depleted cation-adjusted Mueller-Hinton broth.

Results: Of the total 101 isolates, the majority (75, 74.25%) were Enterobacterales which included *Klebsiella pneumonia* (42, 41.58%) and *Escherichia coli* (33, 32.67%), followed by *Pseudomonas aeruginosa* (13, 12.87%) and *Acinetobacter baumannii* (10, 9.9%). Only three (2.97%) of the isolates were *Stenotrophomonas maltophilia*. Most of the isolates were susceptible to cefiderocol, with only four (3.96%) isolates showing resistance. Colistin resistance was observed in six (6.12%) of the isolates. There was a good correlation between disc diffusion testing and broth microdilution testing for the detection of cefiderocol-resistant isolates. No cross-resistance with colistin was observed, as all colistin-resistant isolates were uniformly susceptible to cefiderocol

Conclusion: Cefiderocol is highly effective with good in vitro activity against the majority of carbapenem-resistant pathogens. While some isolates do show resistance, it is relatively uncommon. Given its safety profile compared to colistin, cefiderocol can serve as an alternative to colistin to treat carbapenem-resistant infections and it may be considered even for the management of colistin-resistant cases. Disc diffusion testing is a reliable method for identifying cefiderocol-resistant isolates in routine clinical and diagnostic laboratories, especially in resource-limited settings.

## Introduction

Infectious diseases are among the top leading causes of death. Infections caused by drug-resistant gram-negative bacteria are increasingly being reported. Carbapenem-resistant gram-negative bacteria including *Klebsiella pneumoniae, Acinetobacter baumannii, Pseudomonas aeruginosa, and Enterobacter *spp. are particularly difficult to treat and are considered top-priority pathogens by the World Health Organization [1]. With the emergence of extensively drug-resistant and pan-drug-resistant bacterial infections, which can be resistant to almost all existing groups of antibiotics, including carbapenems, tigecycline, and colistin, the antibiotic pipeline has been left devoid of effective options for treating these infections. Very few new antibiotics have been approved for clinical use in the last two decades. Cefiderocol is a recently introduced novel siderophore cephalosporin, which has structural similarity to cefepime and ceftazidime, is more stable against common mechanisms of beta-lactam resistance and has shown good activity against major drug-resistant pathogens. This stability and enhanced activity are attributed to the presence of a catechol group attached to the C3 side chain, which confers siderophore activity and employs a Trojan horse strategy for uptake via iron transport systems [2-4].

Cefiderocol is highly active against the majority of drug-resistant bacteria, including carbapenem-resistant organisms of *Enterobacteriaceae, Pseudomonas aeruginosa, Acinetobacter baumannii*, and *Stenotrophomonas maltophilia *[5,6]. It has been approved for clinical use in difficult-to-treat bacterial infections caused by cefiderocol-susceptible bacteria. Both disc diffusion and broth microdilution methods are approved by the Clinical and Laboratory Standards Institute (CLSI), with breakpoints available for interpretation. Although broth microdilution is considered the gold standard, it is expensive and tedious to perform as it requires the preparation of iron-depleted, cation-adjusted Mueller-Hinton broth. Disc diffusion is simple, easy, and cost-effective and can be performed on standard Mueller-Hinton agar without the need for iron depletion, making it a viable option in peripheral settings [7,8].

Though cefiderocol is a novel antibiotic with a unique mechanism, resistance to this antibiotic is not uncommon, as evidenced by previous studies in different geographical regions [9]. There are very few studies available regarding the surveillance activity of cefiderocol against carbapenem-resistant gram-negative bacterial isolates from India. It is important to gather surveillance data from various geographical locations to understand the effectiveness of newly approved antibiotics. Thus, this study aims to determine the susceptibility patterns of cefiderocol against various hospital isolates of carbapenem-resistant gram-negative bacterial infections.

## Materials and methods

This observational cross-sectional study included previously characterized and preserved clinical isolates from various clinical specimens received in a 24-hour diagnostic microbiology laboratory at SVS Medical College and Hospital. These isolates were collected between January 2022 and February 2024 and were stored in a -80°C freezer in nutrient/LB broth with 30% glycerol for long-term storage. Approval for the study was obtained from the Institute Ethical Committee (IEC/DHR-04/(04)/2024). A total of 101 carbapenem-resistant gram-negative bacterial isolates were included. Bacterial identification and antibiotic susceptibility testing were conducted using the automated VITEK® 2 Compact identification and susceptibility testing system (bioMérieux SA, Marcy-l'Étoile, France). Cefiderocol susceptibility was assessed for all isolates using disc diffusion testing through the Kirby Bauer method with 30 µg cefiderocol discs (Liofilchem, Teramo, Italy) on standard Muller Hinton agar (HiMedia Laboratories, Mumbai, India). Cefiderocol powder was sourced from Chemscene India Pvt Ltd., Thane, India. For selected isolates (only isolates that were completely resistant or showed intermediate susceptibility by disc diffusion testing), minimum inhibitory concentration (MIC) testing for cefiderocol was conducted using the broth microdilution method with iron-depleted, cation-adjusted Muller Hinton broth. Iron depletion was achieved using Chelex 100 (Nanochemazone, Leduc, Canada) in accordance with the guidelines set by the CLSI. MIC detection was performed in triplicate for each isolate tested (MIC was tested from 0.125 to 32 µg/mL concentration).

Since colistin does not have a disc diffusion testing method and the automated VITEK system only reports intermediate and resistant results, colistin susceptibility was determined using the colistin agar dilution MIC test for *E. coli, Klebsiella pneumoniae* and *Pseudomonas aeruginosa*. This involved using colistin agar plates with concentrations of 0 µg/mL, 1 µg/mL, 2 µg/mL, and 4 µg/mL, following CLSI guidelines [8]. For *Acinetobacter baumannii*, the colistin broth microdilution test was employed, as it is the only CLSI-approved method for this species. Colistin sulfate powder was procured from HiMedia Laboratories. Breakpoints for disc diffusion and MIC were interpreted according to CLSI 2024 guidelines for all antibiotics tested. *Escherichia coli* 25922 and *Pseudomonas aeruginosa* 27853 were used as control strains.

All data were analyzed using Excel (Microsoft, Redmond, WA, USA) and the SPSS statistical package, version 25.0 (IBM Corp., Armonk, NY, USA). Descriptive statistics, including frequency and percentage, were presented in tables, along with visual representations such as bar charts and pie diagrams.

## Results

In the present study, a total of 101 non-duplicate carbapenem-resistant Gram-negative clinical isolates from various clinical samples received in the department of microbiology were included. Most of the isolates were obtained from urine specimens (46, 45.54%), followed by blood (18, 17.82%), pus (17, 16.83%), respiratory specimens (16, 15.84%) and other body fluids (four, 3.96%). Enterobacteriales members, including *Escherichia coli* and *Klebsiella pneumoniae*, were the predominant organisms isolated, constituting three-fourths of the isolates (75, 74.25%). This was followed by *Acinetobacter baumannii *(13, 12.87%) and *Pseudomonas aeruginosa *(10, 9.9%) and* Stenotrophomonas maltophilia* (three, 2.97%) (Table 1).

**Table 1:** Distribution of carbapenem-resistant bacterial isolates in various specimens BAL: Bronchoalveolar lavage

Specimen	Enterobacterales	Acinetobacter baumannii	Pseudomonas aeruginosa	Stenotrophomonas maltophilia
Urine (n=46) (45.54%)	42 (41.58%)	0 (0%)	4 (3.96%)	0 (0%)
Blood (n=18) (17.82%)	11 (10.9%)	4 (3.96%)	1 (0.99%)	2 (1.98%)
Pus/wound swab/tissue (n=17) (16.83%)	13 (12.87%)	1 (0.99%)	3 (2.97%)	0 (0%)
Endotracheal secretions/BAL fluid/sputum (n=16) (15.84%)	7 (6.92%)	6 (5.94%)	2 (1.98%)	1 (0.99%)
Other Body Fluids (n=4) (3.96%)	2 (1.98%)	2 (1.98%)	0 (0%)	0 (0%)
Total = 101 (100%)	75 (74.25%)	13 (12.87%)	10 (9.9%)	3 (2.97%)

**Figure 1:**
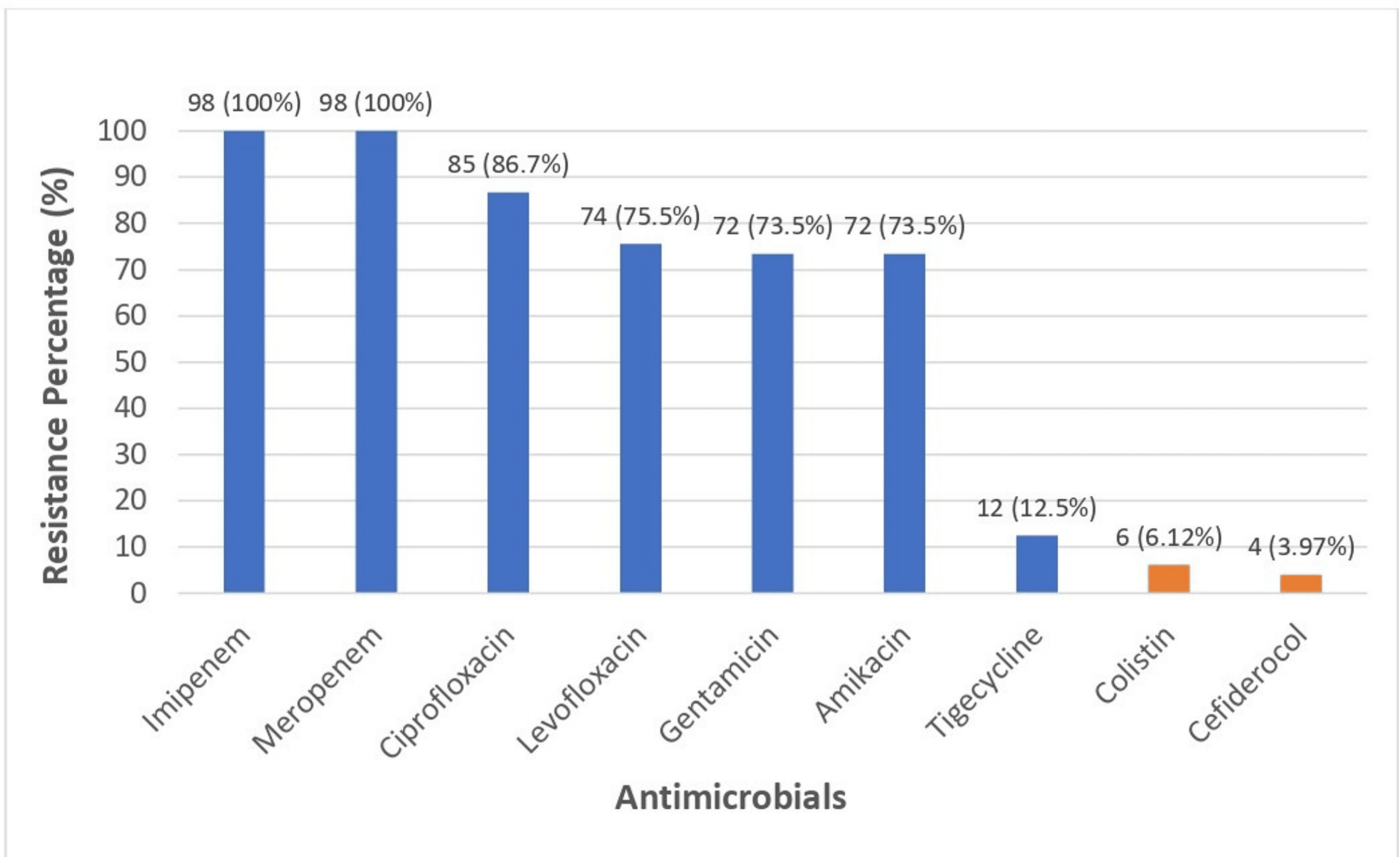
Resistant patterns of the isolates for various antimicrobials The data has been represented as the frequency (n) and percentage (%) of resistance. Antibiotic susceptibility testing was conducted on 98 clinical isolates, excluding *Stenotrophomonas maltophilia*. *Stenotrophomonas maltophilia *was tested solely for cefiderocol and not for other antibiotics

Regarding antibiotic resistance, all the tested isolates were uniformly resistant to carbapenems, including imipenem and meropenem. A high resistance rate was observed for both the fluoroquinolone and aminoglycoside groups. Ciprofloxacin resistance was observed in 85 (86.73%) of the isolates, while levofloxacin resistance was slightly lower at 74 (75.51%). Additionally, 72 (73.5%) of the isolates were resistant to both gentamicin and amikacin. The highest sensitivity was observed for cefiderocol, with only four (3.96%) of isolates being resistant, followed by colistin with six (6.12%) and tigecycline with 12 (12.5%) (Figure 1).

Resistance to cefiderocol was detected in only four out of 101 carbapenem-resistant isolates (3.96%). These resistant isolates were exclusively from Enterobacterales members, including *E. coli* (two, 1.98%) and *K. pneumoniae *(two, 1.98%). No other bacterial species exhibited resistance to cefiderocol. Intermediate susceptibility was observed in three isolates (2.97%), comprising *E. coli *(one, 0.99%)*,* *K. pneumoniae *(one, 0.99%), and *P. aeruginosa *(one, 0.99%) (Table 2).

**Table 2:** Susceptibility profiles for cefiderocol across various bacterial species

Carbapenem Resistant Organism	Total Tested	Cefiderocol		
		Resistant	Intermediate	Susceptible
Escherichia coli	33 (32.67%)	2 (1.98%)	1 (0.99%)	30 (29.70%)
Klebsiella pneumoniae	42 (41.58%)	2 (1.98%)	1 (0.99%)	39 (38.61%)
Acinetobacter baumannii	13 (12.87%)	0 (0%)	0 (0%)	13 (12.87%)
Pseudomonas aeruginosa	10 (9.9%)	0 (0%)	1 (0.99%)	9 (8.91%)
Stenotrophomonas maltophilia	3 (2.97%)	0 (0%)	0 (0%)	3 (2.97%)
Total	101 (100%)	4 (3.96%)	3 (2.97%)	94 (93.07%)

## Discussion

Treating multidrug-resistant pathogens has always been challenging due to the limited therapeutic options available. In India, where there is a significant reservoir of drug-resistant pathogens, particularly Gram-negative bacteria, it is crucial to have effective antibiotics to combat these infections. Cefiderocol holds promise in addressing the gap. Despite its unique mechanism of action and a higher safety profile compared to last-resort drugs like colistin and polymyxin, bacteria can still develop resistance to cefiderocol [9]. Therefore, ongoing surveillance of this drug's efficacy against various drug-resistant pathogens is essential to ensure appropriate treatment and to prevent the emergence of resistance.

The present study assesses cefiderocol susceptibility against major carbapenem-resistant Gram-negative bacteria routinely isolated from clinical specimens. In contrast to broth microdilution methods, disc diffusion testing does not require iron-deficient media. The composition of the media greatly affects the drug susceptibility testing results. Given that the cost of broth microdilution testing for cefiderocol is significantly higher than disc diffusion testing and requires meticulous preparation of iron-deficient cation-adjusted media along with skilled personnel for troubleshooting, it is not practical for routine testing, especially in resource-limited and peripheral settings [8-11]. Therefore, this study primarily utilizes disc diffusion testing, with broth MIC testing using cefiderocol powder (Appendix 1) is reserved only for selected isolates to confirm the results.

In the present study, most isolates were found to be sensitive to this drug, with resistance observed in only four (3.96%) out of 101 isolates. These four resistant isolates were identified by both disc diffusion and broth microdilution methods. Intermediate susceptibility was noted in three additional isolates by disc diffusion testing (Appendix 2), which was confirmed by broth MIC testing. For *Acinetobacter baumannii*, CLSI guidelines do not provide disc diffusion criteria for intermediate and resistant categories. A disc diffusion zone diameter of ≥15 mm is considered sensitive, while a zone diameter ≤14 mm should be interpreted with caution and confirmed by MIC testing [8]. In our study, all but one Acinetobacter baumannii isolate were sensitive by disc diffusion. The exception, with a disc diffusion zone diameter of 11 mm observed in our study, was found to be sensitive by the broth microdilution method (both disc diffusion and broth microdilution were tested in triplicate for this isolate to confirm the results).

All four resistant isolates in the present study were identified within the Enterobacterales group, consisting of two isolates of *E. coli* and two isolates of *Klebsiella pneumoniae*. The MIC for all these resistant isolates exceeded 32 mg/L, as determined by broth microdilution testing. No resistance was observed in other bacteria which include *Pseudomonas aeruginosa, Acinetobacter baumannii *and *Stenotrophmonas maltophilia*. In the intermediate category, disc diffusion testing revealed one isolate of *E. coli*, one isolate of *Klebsiella* *pneumoniae*, and one isolate of *Pseudomonas aeruginosa*. The *Acinetobacter baumannii *isolate, which had a disc diffusion zone size of 11 mm, could not be interpreted with disc diffusion testing. However, it was found to be sensitive when tested using the broth microdilution method.

Numerous studies worldwide have examined the activity of cefiderocol against various drug-resistant Gram-negative bacterial isolates and found good in vitro activity compared to other antibiotics including carbapenems, β-lactam/β-lactamase inhibitor (BL/BLI) combinations, tigecycline and colistin [12,13]. In a study from the SENTRY Antimicrobial Surveillance Program, cefiderocol demonstrated high efficacy against Gram-negative pathogens from hospitalized patients across the U.S. and Europe. Among Enterobacterales, 99.8% were susceptible, including 98.2% of carbapenem-resistant isolates. Cefiderocol demonstrated robust activity against isolates that were resistant to BL/BLI combinations with a high susceptibility rate of 95.1% against isolates resistant to meropenem-vaborbactam and 95.9% against those resistant to imipenem-relebactam and a susceptibility rate of 89.2% against isolates resistant to ceftazidime-avibactam. Cefiderocol also showed 99.6% susceptibility in *P. aeruginosa*, including 97.3% of extensively drug-resistant (XDR) strains, and 97.7% susceptibility in *Acinetobacter *species [12]. Similarly in a study by Delgado-Valverde et al., cefiderocol exhibited strong in vitro activity against the isolates analyzed, with MIC_50_ and MIC_90_ values ranging from 0.125-8 mg/L and 0.5-8 mg/L, respectively. All carbapenemase-producing *K. pneumoniae* strains, including those resistant to ceftazidime/avibactam, were susceptible to cefiderocol. *P. aeruginosa *isolates, including those resistant to ceftolozane/tazobactam, were also susceptible. Moreover, all isolates resistant to colistin remained susceptible to cefiderocol [13].

Currently, there are only a limited number of studies from India on cefiderocol susceptibility testing [14-16]. Only two studies from India have included disc diffusion testing for cefiderocol in their research. Our study results are similar to those of Khanchanadani et al., who also used disc diffusion testing [15]. In their study, 93.3% of Enterobacteriaceae isolates were susceptible to cefiderocol, and 100% of *Acinetobacter baumannii *were susceptible. While they observed one resistant isolate of *Pseudomonas aeruginosa*, all our *Pseudomonas* isolates were susceptible, with one falling into the intermediate category. In a single-center study with a large number of isolates conducted by Nayak et al., 81.3% of carbapenem-resistant Enterobacterales were susceptible to cefiderocol, while 82.6% of non-fermenting Gram-negative bacilli were susceptible [16]. They utilized both disc diffusion and broth microdilution methods, conducting a comparative analysis to determine categorical agreement. Their study also included molecular characterization of drug-resistant genes. In their findings, 8.7% of the isolates were resistant by disc diffusion testing, and 10.2% were intermediate, which is higher than our results. These differences may be partially attributed to changes in the CLSI interpretative breakpoints observed over the past three years [8,15].

In the present study, colistin resistance was detected in four out of 101 isolates using the VITEK 2 system, with all other isolates classified as intermediate. However, agar MIC/broth microdilution testing identified a total of six isolates as colistin-resistant. Among these, four were *Klebsiella pneumoniae* and two were *Acinetobacter baumannii*. Notably, all six colistin-resistant isolates were susceptible to cefiderocol, showing no cross-resistance with colistin. This suggests that cefiderocol could be an effective treatment option for infections caused by these colistin-resistant isolates.

Limitations of the study

There are some limitations to this study. It was conducted at a single center and included a limited number of isolates. Additionally, broth microdilution was not performed for all isolates due to the high cost and complexity of the procedure. Furthermore, the study did not include molecular characterization to identify the mechanisms of carbapenem resistance in the clinical isolates.

## Conclusions

Cefiderocol has demonstrated good in vitro activity against most carbapenem-resistant Gram-negative clinical isolates. However, resistance was noted in some isolates of Enterobacteriaceae, whereas non-fermenting Gram-negative bacteria were uniformly susceptible to cefiderocol. Disc diffusion is a practical and reliable method for assessing cefiderocol susceptibility in routine diagnostic laboratories, especially in resource-limited settings.

## Figures and Tables

**Figure 2:**
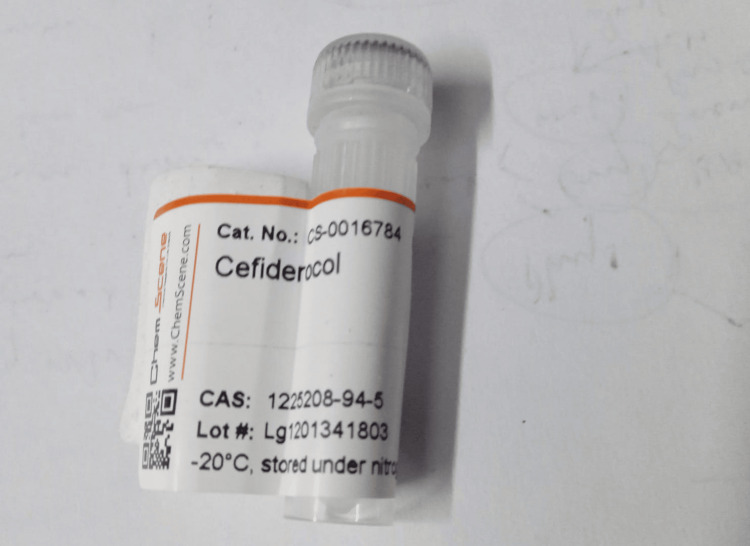
Pure powder of cefiderocol used for broth microdilution testing

**Figure 3:**
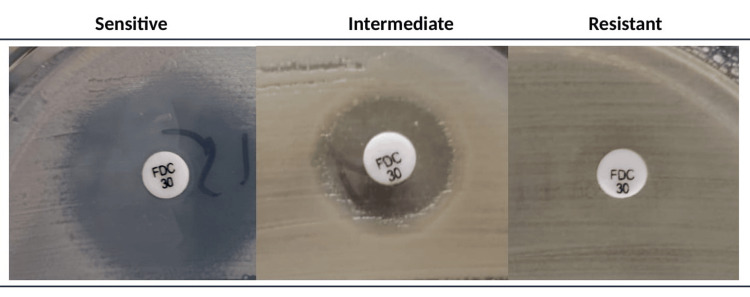
Sensitive, intermediate and resistant isolates of cefiderocol by disc diffusion

## Appendices

Appendix 1

Appendix 2
